# Relationship between spending on electronic cigarettes, 30-day use, and disease symptoms among current adult cigarette smokers in the U.S.

**DOI:** 10.1371/journal.pone.0187399

**Published:** 2017-11-07

**Authors:** Tingting Yao, Wendy Max, Hai-Yen Sung, Stanton A. Glantz, Rachel L. Goldberg, Julie B. Wang, Yingning Wang, James Lightwood, Janine Cataldo

**Affiliations:** 1 Institute for Health & Aging, School of Nursing, University of California, San Francisco, CA, United States of America; 2 Center for Tobacco Control Research and Education, Cardiovascular Research Institute, School of Medicine, University of California, San Francisco, CA, United States of America; 3 Department of Physiological Nursing, School of Nursing, University of California, San Francisco, CA, United States of America; 4 Department of Clinical Pharmacy, School of Pharmacy, University of California, San Francisco, CA, United States of America; University of Cape Town Lung Institute, SOUTH AFRICA

## Abstract

**Objective:**

To examine the relationship between spending on electronic cigarettes (e-cigarettes) and disease symptoms compared with the relationship between 30-day e-cigarette use and disease symptoms among adult cigarette smokers in the U.S.

**Methods:**

We analyzed data from the Tobacco and Attitudes Beliefs Survey which included 533 respondents aged 24+ who were current cigarette smokers and e-cigarette ever users. Fifteen self-reported disease symptoms were included as outcome variables. Separate multivariable logistic regression models were estimated for each disease symptom with total spending on e-cigarettes in the past 30 days and with reported 30-day e-cigarette use. All models controlled for cigarettes smoked per day (CPD) and sociodemographic characteristics.

**Results:**

We found that those who spent more on e-cigarettes were more likely to report chest pain (AOR = 1.25, 95% CI 1.02–1.52), to notice blood when brushing their teeth (AOR = 1.23, 95% CI 1.02–1.49), to have sores or ulcers in their mouth (AOR = 1.36, 95% CI 1.08–1.72), and to have more than one cold (AOR = 1.36, 95% CI 1.05–1.78) than those with no spending on e-cigarettes in the past 30 days in an adjusted analysis. After controlling for CPD and other covariates, there were no significant relationships between 30-day e-cigarette use and symptoms. Even after controlling for CPD, e-cigarette expenditures or use was associated with greater odds of wheezing and shortness of breath.

**Conclusions:**

E-cigarette expenditures might be a more useful measure of intensity of e-cigarette use. The additional health effect of e-cigarette use or expenditures among smokers independent of the effect of CPD suggests that e-cigarette use adds adverse health effects even among cigarette smokers.

## Introduction

In recent years, electronic cigarette (e-cigarette) use has substantially increased among U.S. adults, but the intensity of e-cigarette use is difficult to measure. National Health Interview Survey (NHIS) data show that the prevalence of having ever tried an e-cigarette among U.S. adults increased from 8.1% in 2012 to 12.6% in 2014 [1–2]. Earlier data from the U.S. Centers for Disease Control and Prevention (CDC) found that in 2013, 8.5% of adults had used an e-cigarette at least once in their life and the prevalence of past 30-day e-cigarette use among adults increased from 1% in 2010 to 2.6% in 2013 [3]. Data from the 2015 NHIS show that 3.5% of adults currently use e-cigarettes every day or some days [4].

Most current adult and youth e-cigarette users are dual users of e-cigarettes and conventional cigarettes [5]. A CDC report showed that 59% of adult e-cigarette users and 76.3% of youth e-cigarette users are dual users [6]. Data from the 2014 NHIS indicate that 15.9% of adult current cigarette smokers also concurrently use e-cigarettes [7]. The dual use of e-cigarettes and cigarettes raises concerns beyond the potential health effects of using e-cigarettes alone.

E-cigarettes have been marketed as helping smokers quit conventional cigarette smoking, being less harmful than conventional cigarettes, and creating no secondhand smoke [8,9], but many questions remain about the potential risks to public health posed by e-cigarette products. There is not sufficient research on the health effects of using e-cigarettes [10]. According to the CDC, e-cigarette aerosol is not harmless “water vapor” [11], and can contain harmful and potentially harmful ingredients, including heavy metals, ultrafine particulate matter, and cancer-causing agents like acrolein [12]. The U.S. Food and Drug Administration (FDA) has analyzed e-cigarette cartridges and found them to contain disthylene glycol (a known toxicant when inhaled), and also discovered inaccuracies in labeling nicotine concentrations [13,14]. A study conducted in 2014 examined the online market for e-cigarettes and found that there were 466 brands and 7,764 different flavors of e-cigarette products [15]. This further complicates research on potential health effects of e-cigarette use, as each brand and flavor combination can yield unique outcomes. E-cigarette manufacturers claim that the use of food flavorings is safe because they meet the FDA’s definition of “Generally Recognized as Safe” (GRAS), but the GRAS status applies to additives for use in foods (ingestion), not for inhalation. Inhaling many of these substances has been shown to have negative health effects [11].

Several studies have examined short-term health symptoms associated with e-cigarette use. Cross-sectional studies of children found a positive association between e-cigarette use and (1) self-reported respiratory symptoms (cough or phlegm)[16] in 7th-12th grade students in Hong Kong and (2) asthma [17] in high school students in South Korea. Among Southern California 11th-12th grade adolescents, e-cigarette use was positively associated with self-reported chronic bronchitis symptoms but after adjusting for cigarette use, there was no association with self-reported wheezing [18]. For adults, studies have reported harmful effects of e-cigarette use on pulmonary function [19,20]. A systematic review of 76 studies on health consequences of e-cigarettes use among adults concluded that short-term adverse effects of airway resistance and inflammation occur among e-cigarette users [21]. In addition, a recent study found a positive association of current e-cigarette use with asthma and cardiovascular disease among current cigarette smokers with medical comorbidities [22].

Cigarette use intensity and frequency are usually summarized by the average number of cigarettes smoked per day, but measurement of e-cigarette use is complicated by the fact that 1) there are hundreds of different product types (e.g., disposable e-cigarettes, reusable/refillable/rechargeable e-cigarettes), which differ in the temperature and power that is applied; 2) the nicotine content in e-cigarette liquid varies widely; and 3) daily use patterns vary, which makes it challenging to develop a common “unit” of use that can easily summarize frequency or intensity of typical e-cigarette use [23–25]. Spending on e-cigarettes might be a useful proxy for intensity and frequency of e-cigarette use because measuring use in dollar terms allows the different products and use patterns to be measured in a common metric. Spending on e-cigarettes is associated with e-cigarette use. A study of Canadian 9th-12th grade students found that students who spent more money on e-cigarettes were more likely to report past-month e-cigarette use (yes/no) [26].

This study examines the relationship between spending on e-cigarettes and disease symptoms compared with the relationship between 30-day e-cigarette use and disease symptoms among current U.S. adult cigarette smokers. We hypothesize that e-cigarette expenditures is a better measure for analysis of correlation with symptoms because it incorporates both the different types of products and the intensity of use into a single measure.

## Methods

### Data source

We analyzed data from the first wave of the longitudinal Tobacco and Attitudes Beliefs Survey (TABS), which was conducted with a Qualtrics panel of US adults from August 20–31, 2015. The TABS survey was adapted from previous survey questions on e-cigarette use, perceptions of risks, and symptom experience that have been previously validated and used with adolescents, young adults, and adults [27–29]. The TABS survey was piloted on a small cohort of adolescent current and former smokers prior to dissemination [30,31]. This survey investigated participants’ tobacco use experiences; exposure to pro- and anti-tobacco media messages; perceptions of tobacco-product acceptability, risks, and benefits; and both intention to use and actual use of tobacco products. The survey also included questions on e-cigarette spending and disease symptoms. Participants were recruited by the Qualtrics Research Company (an online survey company) using a probability-based sampling approach. Participants received $10 for completing the survey. All procedures were approved by the University of California, San Francisco Institutional Review Board. Written informed consent was obtained prior to data collection. A total of 819 participants who were aged 24 years and older completed the first wave of the TABS survey, including 716 current cigarette smokers (those who smoked cigarettes less than 5 years ago) and 103 former cigarette smokers (quit smoking less than five years ago). Among the 819 participants, 610 of them were e-cigarette ever users and 288 of them were current (last 30 days) e-cigarette users.

### Dependent variables

In TABS, participants who ever used e-cigarettes were asked about their disease symptoms in the past 30 days. Fifteen disease symptoms were examined: coughing, wheezing, shortness of breath, chest tightness, headache, sore throat, waking up feeling tired, chest pain, having trouble falling asleep or staying asleep, toothache, sensitive teeth, noticing blood when brushing their teeth, having sores or ulcers in their mouth, having one cold, and having more than one cold. These symptoms were selected because they were reported by e-cigarette users in a previous study [29]. Respondents were asked “Did you have any of the following symptoms in the past 30 days?” For each symptom, participants were assigned the value of 1 for “yes” and 0 for “no”.

### Independent variables

#### Current (last 30 days) e-cigarette use

Current e-cigarette use was measured by a dichotomous (yes/no) variable based on the answer to the question: “Have you used e-cigarettes in the last 30 days?”

#### Total spending on e-cigarettes in the last month

The variable for total spending on e-cigarettes was constructed from three questions. The first question was, “Have you ever bought an e-cigarette (e-cig, vaporizer, vapor pen, vape or mod) or its components?” Participants who answered “yes” were then asked: “What type of e-cigarettes (e-cig, vaporizer, vapor pen, vape or mod) or its components did you buy last time?” Response options were starter kit, disposable e-cigarettes, refillable/rechargeable/reusable e-cigarettes, e-juice (also called e-liquid), accessories (i.e., chargers, atomizers, batteries), and other type (describe). For each type of e-cigarette the participants chose, they were asked a third question: “How much (US dollars, including shipping and tax) did you spend for the type of e-cigarettes you chose in the second question in the last month?” Because participants could buy more than one type of e-cigarette, the total spending on e-cigarettes was obtained by summing the respondent’s spending on all types of e-cigarettes purchased last month.

#### Average number of conventional cigarettes smoked per day (CPD)

CPD was measured by the question: “On average, how many cigarettes a day do you smoke?”

#### Sociodemographic characteristics

Sociodemographic variables included age (24–45, 45–64, and 65+ years old), gender (male and female), race/ethnicity (Non-Hispanic [NH] African-American, NH Asian, NH White, Hispanic, and NH Other), education (less than high school degree, completed high school (including General Educational Development certificate), some college, and college degree and above), occupational status (employed, unemployed, retired, full-time homemaker, and student), yearly personal income (<$30,000, $30,000-$49,999, $50,000–$99,999, and ≥$100,000), and marital status (married, partnered, divorced/separated/widowed, and single/never married).

### Study sample

Because only ever users of e-cigarettes were asked about disease symptoms in the past 30 days, the study sample was comprised of current cigarette smokers who were e-cigarettes ever users (N = 539). Among the 539 participants, 262 of them were current (last 30 days) e-cigarette users. After excluding 6 participants with missing information on CPD (N = 3, 0.6%) or race/ethnicity (N = 3, 0.6%), the final study sample consisted of 533 participants.

### Statistical analysis

Multivariable logistic regression analyses were used to analyze the factors associated with having disease symptoms using 2 alternative measures of e-cigarette use. One set of models included total spending on e-cigarettes in the last month and another set included current (30 day) e-cigarette use. Separate logistic regressions were estimated for each of the 15 symptoms. All models controlled for CPD and sociodemographic characteristics. All analyses were carried out using SAS version 9.4 (SAS Institute, Cary, NC). A two-tailed p-value <0.05 was considered to be statistically significant.

## Results

### Sociodemographic and tobacco use characteristics

The sample distribution by sociodemographic characteristics is shown in Table 1. Among the final study sample, 80.5% were NH White; slightly more than half were aged 45–64, female, or employed, nearly half had an annual income of <$30,000 or were married, and 30.4% had less than a high school education. In the past 30 days, 48.8% of participants had used e-cigarettes and 44.5% had purchased e-cigarettes. Mean CPD was 15.4 (normally distributed; SD = 1.00) and the mean expenditures on e-cigarettes (not normally distributed) was $40.0 (Interquartile range: 25 percentile = $0 and 75 percentile = $25.0), respectively.

**Table 1:** Demographic characteristics of study participants (N = 533).

	N	Column %
Total	533	100.0
**Age**		
24–44	199	37.3
45–64	295	55.4
≥65	39	7.3
**Gender**		
Male	217	40.7
Female	316	59.3
**Race and ethnicity**		
NH African American	35	6.6
NH Asian	9	1.7
NH Others	15	2.8
NH White	429	80.5
Hispanic	45	8.4
**Education (highest level completed)**		
Less than high school	162	30.4
Complete HS	200	37.5
Some college	134	25.1
College+	37	6.9
**Occupation status**		
Employed	285	53.5
Unemployed	70	13.1
Retired	100	18.8
Full-time homemaker	71	13.3
Student	7	1.3
**Yearly personal income**		
$0–$30K	257	48.2
$31k-$50k	114	21.4
$51k-$100k	137	25.7
> $100k	25	4.7
**Marital status**		
Married	254	47.7
Partnered	47	8.8
Divorced/Separated/Widowed	136	25.5
Single, never married	96	18.0
**Current (30 day) e-cigarette user**		
No	273	51.2
Yes	260	48.8
**CPD (mean = 15.4)**		
0–9	131	24.6
10–19	204	38.3
> = 20	198	37.2
**Total e-cigarette expenditure last month (mean = $40)**		
No (= 0)	296	55.5
Yes (>0)	237	44.5

### Prevalence of disease symptoms

Table 2 shows that the most commonly reported disease symptoms were coughing (54.5%) and waking up feeling tired (52.0%), and the least common symptoms were having sores or ulcers in mouth (8.3%) and having more than one cold (6.8%).

**Table 2:** Disease symptoms rates (N = 533).

	Disease symptom rates (%)
Coughing	54.8
Wheezing	32.1
Shortness of breath	41.8
Chest tightness	22.5
Headache	44.1
Sore throat	19.7
Woke up feeling tired	52.0
Chest pains	9.9
Had trouble falling asleep	48.2
Toothache	16.7
Sensitive teeth	29.1
Noticed blood when brush teeth	17.1
Had sores or ulcers in mouth	8.3
Had one cold	14.8
Had more than one cold	6.8

### Factors associated with disease symptoms

Table 3 shows the results of the multivariable logistic regression models for disease symptoms with total e-cigarette expenditures, CPD, and sociodemographic characteristics as other covariates. For each $100 increase in e-cigarette expenditures, the odds of reporting chest pain (versus not reporting chest pain), noticing blood when brushing teeth (versus not noticing blood when brushing teeth), having sores or ulcers in their mouth (versus not having sores or ulcers in their mouth), and having more than one cold (versus not having more than one cold) significantly increased by factors of 1.25, 1.23, 1.36, and 1.36, respectively. Similarly to the model presented in Table 3, for each 10 cigarettes smoked per day, the odds of reporting wheezing (versus not reporting wheezing) and shortness of breath (versus not reporting shortness of breath) increased by factors of 1.38 and 1.24, respectively.

**Table 3:** Estimated multivariable logistic regression model (OR and 95% CI) on disease symptoms controlling for e-cigarette expenditure, CPD, and sociodemographic characteristics (N = 533).

	Coughing	Wheezing	Shortness of breath	Chest tightness	Headache	Sore throat	Woke up feeling tired	Chest pains	Had trouble falling asleep	Toothache	Sensitive teeth	Noticed blood when brush teeth	Had sores or ulcers in mouth	Had one cold	Had more than one cold
	AOR (95% CI)	AOR (95% CI)	AOR (95% CI)	AOR (95% CI)	AOR (95% CI)	AOR (95% CI)	AOR (95% CI)	AOR (95% CI)	AOR (95% CI)	AOR (95% CI)	AOR (95% CI)	AOR (95% CI)	AOR (95% CI)	AOR (95% CI)	AOR (95% CI)
**Total e-cigarette expenditure last month (per $100)**	1.11 (0.93,1.32)	1.10 (0.95, 1.26)	0.98 (0.86, 1.11)	0.93 (0.80, 1.08)	0.98 (0.87, 1.11)	1.02 (0.90, 1.16)	0.92 (0.78, 1.09)	**1.25** (1.02, 1.52)	0.97 (0.81, 1.15)	1.07 (0.94, 1.22)	1.05 (0.92, 1.20)	**1.23** (1.02, 1.49)	**1.36** (1.08, 1.72)	1.03 (0.90, 1.18)	**1.36** (1.05, 1.28)
**CPD (per 10 CPD)**	1.15 (0.95, 1.39)	**1.38** (1.12, 1.69)	**1.24** (1.02, 1.50)	1.16 (0.94, 1.45)	0.97 (0.80, 1.17)	1.06 (0.83, 1.35)	1.17 (0.96, 1.43)	1.19 (0.84, 1.66)	1.17 (0.96, 1.43)	0.96 (0.72, 1.29)	0.85 (0.67, 1.07)	0.74 (0.53, 1.03)	1.27 (0.85, 1.91)	0.80 (0.57, 1.12)	0.65 (0.32, 1.31)
**Age**															
<45 (Ref)															
45–64	1.30 (0.83, 2.02)	0.99 (0.62, 1.60)	0.81 (0.52, 1.25)	0.69 (0.41, 1.15)	**0.62** (0.40, 0.95)	**0.49** (0.29, 0.83)	1.18 (0.77, 1.83)	0.58 (0.29, 1.16)	**1.58** (1.01, 2.46)	0.70 (0.40, 1.24)	**0.54** (0.34, 0.87)	**0.49** (0.27, 0.90)	**0.37** (0.15, 0.89)	**0.41** (0.22, 0.77)	**0.27** (0.09, 0.86)
≥65	1.85 (0.76, 4.52)	1.05 (0.42, 2.62)	1.11 (0.47, 2.62)	**0.32** (0.10, 0.99)	**0.19** (0.07, 0.56)	0.31 (0.08, 1.27)	**0.37** (0.15, 0.91)	<0.001 (<0.001, >999.99)	0.70 (0.29, 1.70)	<0.001 (<0.001, >999.99)	**0.13** (0.03, 0.49)	**0.10** (0.01, 0.85)	0.33 (0.03, 3.22)	0.42 (0.10, 1.78)	<0.001 (<0.001, >999.99)
**Gender**															
Male (Ref)															
Female	1.00 (0.67, 1.50)	1.09 (0.71, 1.68)	0.92 (0.62, 1.38)	1.21 (0.75, 1.97)	**1.69** (1.12, 2.54)	0.88 (0.52, 1.48)	**1.64** (1.10, 2.44)	0.84 (0.44, 1.61)	1.33 (0.89, 1.98)	1.11 (0.64, 1.92)	1.06 (0.68, 1.65)	**0.48** (0.27, 0.84)	0.45 (0.18, 1.08)	0.71 (0.39, 1.28)	0.41 (0.13, 1.36)
**Race and ethnicity**															
NH African American (Ref)															
NH Asian	1.26 (0.21, 7.79)	0.59 (0.06, 5.79)	2.23 (0.45, 11.09)	<0.001 (<0.001, >999.99)	0.43 (0.07, 2.60)	0.98 (0.08, 11.72)	1.95 (0.40, 9.60)	1.45 (0.12, 17.68)	1.52 (0.31, 7.36)	0.71 (0.07, 7.75)	2.92 (0.56, 15.19)	0.50 (0.05, 5.21)	<0.001 (<0.001, >999.99)	1.31 (0.18, 9.42)	<0.001 (<0.001, >999.99)
NH Others	1.87 (0.51, 6.88)	0.66 (0.14, 3.12)	0.53 (0.13, 2.10)	<0.001 (<0.001, >999.99)	1.29 (0.35, 4.72)	<0.001 (<0.001, >999.99)	1.15 (0.32, 4.14)	<0.001 (<0.001, >999.99)	0.57 (0.16, 2.05)	0.90 (0.20, 3.94)	1.94 (0.48, 7.82)	<0.001 (<0.001, >999.99)	<0.001 (<0.001, >999.99)	<0.001 (<0.001, >999.99)	8.22 (0.32, 210.03)
NH White	**2.84** (1.30, 6.18)	1.17 (0.50, 2.73)	0.92 (0.44, 1.94)	0.99 (0.40, 2.47)	1.31 (0.61, 2.83)	2.63 (0.75, 9.26)	1.11 (0.52, 2.36)	0.85 (0.26, 2.75)	0.91 (0.44, 1.92)	0.53 (0.21, 1.31)	1.44 (0.60, 3.48)	0.53 (0.21, 1.35)	1.34 (0.16,11.39)	1.00 (0.35, 2.90)	0.61 (0.06, 5.88)
Hispanic	**7.14** (2.40, 21.28)	**3.18** (1.07, 9.43)	2.21 (0.80, 6.10)	2.31 (0.73, 7.28)	1.91 (0.68, 5.37)	**5.54** (1.30, 23.67)	0.53 (0.19, 1.51)	**0.04** (0.00, 0.90)	**0.23** (0.07, 0.72)	0.64 (0.19, 2.16)	2.70 (0.89, 8.18)	1.11 (0.34, 3.59)	4.04 (0.40,41.00)	3.09 (0.88,10.94)	2.36 (0.21, 27.05)
**Education (highest level completed)**															
Less than high school (Ref)															
Complete HS	0.71 (0.45, 1.12)	0.70 (0.44, 1.13)	0.70 (0.45, 1.09)	0.66 (0.39, 1.12)	**0.62** (0.39, 0.97)	0.61 (0.34, 1.10)	0.77 (0.49, 1.22)	0.98 (0.48, 2.00)	0.90 (0.57, 1.41)	0.87 (0.47, 1.60)	0.66 (0.40, 1.08)	0.79 (0.43, 1.45)	**0.35** (0.15, 0.86)	0.78 (0.41, 1.50)	0.82 (0.26, 2.58)
Some college	0.88 (0.52, 1.49)	0.67 (0.39, 1.17)	**0.53** (0.32, 0.90)	0.82 (0.45, 1.49)	0.78 (0.46, 1.32)	0.95 (0.51, 1.78)	0.74 (0.44, 1.26)	0.94 (0.39, 2.26)	1.10 (0.64, 1.89)	1.36 (0.70, 2.64)	0.70 (0.40, 1.22)	0.49 (0.23, 1.04)	**0.09** (0.02, 0.41)	0.70 (0.34, 1.46)	**0.14** (0.03, 0.65)
College+	**0.23** (0.10, 0.55)	**0.29** (0.11, 0.79)	**0.30** (0.12, 0.73)	0.53 (0.18, 1.55)	0.74 (0.33, 1.67)	0.84 (0.31, 2.29)	0.60 (0.27, 1.37)	0.40 (0.05, 3.46)	0.62 (0.26, 1.45)	0.44 (0.09, 2.14)	**0.35** (0.13, 0.96)	0.70 (0.22, 2.23)	0.19 (0.03, 1.04)	0.47 (0.13, 1.63)	0.20 (0.03, 1.48)
**Occupation status**															
Employed (Ref)															
Unemployed	1.54 (0.84, 2.85)	**2.02** (1.06, 3.83)	1.65 (0.90, 3.02)	1.80 (0.88, 3.68)	1.43 (0.78, 2.60)	0.96 (0.42, 2.20)	0.97 (0.53, 1.78)	1.13 (0.43, 2.96)	1.02 (0.56, 1.85)	**3.06** (1.45, 6.47)	1.73 (0.90, 3.33)	1.14 (0.50, 2.59)	0.96 (0.25, 3.78)	1.71 (0.69, 4.27)	1.73 (0.24, 12.70)
Retired	1.50 (0.84, 2.69)	**1.95** (1.05, 3.60)	**1.91** (1.07, 3.40)	**2.03** (1.01, 4.06)	0.78 (0.42, 1.43)	0.53 (0.21, 1.33)	0.98 (0.55, 1.75)	1.48 (0.59, 3.69)	0.90 (0.50, 1.59)	1.22 (0.50, 2.96)	1.65 (0.84, 3.22)	0.64 (0.26, 1.59)	0.47 (0.10, 2.11)	1.26 (0.48, 3.30)	0.98 (0.09, 10.40)
Full-time homemaker	**1.95** (1.05, 3.63)	1.15 (0.61, 2.19)	1.46 (0.80, 2.67)	1.09 (0.55, 2.19)	1.65 (0.91, 3.02)	1.46 (0.72, 2.97)	1.33 (0.73, 2.43)	1.09 (0.42, 2.85)	**3.03** (1.60, 5.73)	**2.29** (1.09, 4.81)	1.27 (0.66, 2.43)	0.82 (0.34, 2.00)	0.86 (0.26, 2.90)	1.40 (0.60, 3.27)	1.83 (0.40, 8.37)
Student	2.34 (0.47, 11.79)	1.30 (0.23, 7.52)	1.49 (0.30, 7.39)	0.63 (0.07, 5.66)	1.41 (0.29, 6.88)	0.51 (0.05, 4.86)	2.06 (0.37, 11.57)	<0.001 (<0.001, >999.99)	1.23 (0.24, 6.16)	2.47 (0.41, 14.74)	1.81 (0.34, 9.52)	2.94 (0.50, 17.17)	**8.17** (1.02, 65.23)	1.11 (0.11,10.88)	<0.001 (<0.001, >999.99)
**Yearly personal income**	** **	** **	** **	** **	** **	** **	** **	** **	** **	** **	** **	** **	** **	** **	** **
$0–$30K (Ref)															
$31k-$50k	0.66 (0.40, 1.09)	1.02 (0.59, 1.78)	0.96 (0.58, 1.60)	1.34 (0.74, 2.42)	1.07 (0.64, 1.77)	1.67 (0.89, 3.16)	1.42 (0.86, 2.36)	0.91 (0.40, 2.06)	0.91 (0.55, 1.51)	1.30 (0.67, 2.53)	1.38 (0.79, 2.41)	0.74 (0.36, 1.53)	0.55 (0.17, 1.82)	0.70 (0.31, 1.57)	4.41 (0.97, 19.93)
$51k-$100k	0.99 (0.57, 1.72)	1.66 (0.93, 2.97)	1.45 (0.84, 2.51)	1.45 (0.76, 2.76)	1.04 (0.60, 1.80)	1.57 (0.78, 3.15)	1.03 (0.60, 1.77)	1.04 (0.43, 2.50)	1.00 (0.57, 1.73)	1.42 (0.69, 2.95)	1.63 (0.89, 2. 99)	0.86 (0.40, 1.87)	1.12 (0.34, 3.64)	1.24 (0.56, 2.73)	**7.96** (1.67, 38.10)
> $100k	0.35 (0.12, 1.03)	0.64 (0.18, 2.28)	0.58 (0.19, 1.74)	0.47 (0.09, 2.36)	0.96 (0.36, 2.56)	1.55 (0.47, 5.18)	0.66 (0.25, 1.76)	<0.001 (<0.001, >999.99)	0.45 (0.16, 1.26)	<0.001 (<0.001, >999.99)	1.34 (0.44, 4.09)	0.40 (0.08, 2.06)	0.71 (0.07, 6.79)	1.40 (0.37, 5.24)	<0.001 (<0.001, >999.99)
**Marital status**	** **	** **	** **	** **	** **	** **	** **	** **	** **	** **	** **	** **	** **	** **	** **
Married (Ref)															
Partnered	1.23 (0.62, 2.44)	1.42 (0.71, 2.85)	1.37 (0.70, 2.68)	1.02 (0.46, 2.23)	1.12 (0.57, 2.23)	1.55 (0.66, 3.64)	0.98 (0.50, 1.92)	1.57 (0.59, 4.15)	1.37 (0.69, 2.71)	1.11 (0.46, 2.66)	1.31 (0.62, 3.83)	1.54 (0.62, 3.83)	0.72 (0.18, 2.86)	1.10 (0.42, 2.84)	<0.001 (<0.001, >999.99)
Divorced/Separated/Widowed	0.91 (0.56, 1.49)	0.92 (0.54, 1.55)	1.20 (0.73, 1.97)	1.09 (0.62, 1.94)	1.20 (0.72, 1.98)	1.79 (0.95, 3.39)	1.12 (0.68, 1.82)	1.10 (0.50, 2.45)	1.35 (0.83, 2.21)	1.40 (0.72, 2.70)	1.48 (0.85, 2.57)	**2.28** (1.14, 4.54)	0.79 (0.27, 2.31)	0.71 (0.32, 1.57)	0.60 (0.11, 3.20)
Single, never married	1.00 (0.58, 1.72)	0.87 (0.48, 1.57)	1.21 (0.70, 2.09)	0.63 (0.32, 1.26)	1.04 (0.60, 1.80)	0.94 (0.45, 1.94)	**2.12** (1.22, 3.68)	0.83 (0.34, 2.03)	1.57 (0.91, 2.70)	0.82 (0.39, 1.71)	1.35 (0.75, 2.43)	0.80 (0.37, 1.76)	**0.23** (0.06, 0.91)	0.66 (0.29, 1.47)	0.54 (0.12, 2.44)

Note: Bold numbers represent p<0.05

Table 4 shows the results of the multivariable logistic regression models for disease symptoms with 30-day e-cigarette use, CPD and sociodemographic characteristics as other covariates. No statistically significant associations were found between 30-day e-cigarette use and any disease symptoms. For each 10 cigarettes smoked per day, the odds of reporting wheezing (versus not reporting wheezing) and shortness of breath (versus not reporting shortness of breath) increased by factors of 1.34 and 1.23, respectively.

**Table 4:** Estimated multivariable logistic regression model on disease symptoms (OR and 95% CI) controlling for 30-day e-cigarette use, CPD, and sociodemographic characteristics (N = 533).

	Coughing	Wheezing	Shortness of breath	Chest tightness	Headache	Sore throat	Woke up feeling tired	Chest pains	Had trouble falling asleep	Toothache	Sensitive teeth	Noticed blood when brush teeth	Had sores or ulcers in mouth	Had one cold	Had more than one cold
	AOR (95% CI)	AOR (95% CI)	AOR (95% CI)	AOR (95% CI)	AOR (95% CI)	AOR (95% CI)	AOR (95% CI)	AOR (95% CI)	AOR (95% CI)	AOR (95% CI)	AOR (95% CI)	AOR (95% CI)	AOR (95% CI)	AOR (95% CI)	AOR (95% CI)
**Current (30 day) e-cigarette user**	** **	** **	** **	** **	** **	** **	** **	** **	** **	** **	** **	** **	** **	** **	** **
No (Ref)	** **	** **	** **	** **	** **	** **	** **	** **	** **	** **	** **	** **	** **	** **	** **
Yes	0.86 (0.59, 1.26)	0.71 (0.48, 1.07)	0.81 (0.55, 1.18)	0.89 (0.57, 1.38)	0.98 (0.67, 1.42)	0.96 (0.59, 1.54)	0.56 (0.38, 1.82)	0.51 (0.27, 1.96)	0.58 (0.40, 0.85)	0.84 (0.51, 1.39)	0.93 (0.61, 1.40)	1.02 (0.60, 1.72)	1.37 (0.63, 3.00)	0.76 (0.43, 1.32)	1.59 (0.58, 4.36)
**CPD (per 10 CPD)**	1.14 (0.94, 1.38)	**1.34** (1.10, 1.65)	**1.23** (1.01, 1.49)	1.16 (0.93, 1.44)	0.97 (0.80, 1.17)	1.06 (0.83, 1.35)	1.15 (0.95, 1.40)	1.07 (0.76, 1.50)	1.15 (0.94, 1.40)	0.94 (0.70, 1.26)	0.84 (0.66, 1.07)	0.73 (0.52, 1.02)	1.26 (0.85, 1.88)	0.78 (0.56, 1.10)	0.63 (0.32, 1.26)
**Age**															
<45 (Ref)															
45–64	1.24 (0.79, 1.93)	0.93 (0.58, 1.49)	0.79 (0.51, 1.23)	0.70 (0.42, 1.17)	**0.62** (0.40, 0.96)	**0.48** (0.29, 0.82)	1.14 (0.74, 1.77)	0.51 (0.26, 1.01)	1.51 (0.97, 2.35)	0.68 (0.39, 1.18)	**0.53** (0.33, 0.84)	**0.46** (0.26, 0.83)	**0.33** (0.14, 0.77)	**0.40** (0.21, 0.74)	**0.25** (0.08, 0.75)
≥65	1.69 (0.69,4.13)	0.91 (0.37, 2.28)	1.05 (0.44, 2.51)	0.33 (0.11, 1.00)	**0.19** (0.07, 0.56)	**0.30** (0.07, 1.24)	**0.32** (0.13, 0.80)	<0.001 (<0.001, >999.99)	0.61 (0.25, 1.49)	<0.001 (<0.001, >999.99)	**0.12** (0.03, 0.46)	**0.09** (0.01, 0.75)	0.29 (0.03, 2.88)	0.38 (0.09, 1.62)	<0.001 (<0.001, >999.99)
**Gender**															
Male (Ref)															
Female	1.03 (0.69, 1.54)	1.13 (0.73, 1.75)	0.93 (0.62, 1.39)	1.20 (0.74, 1.95)	**1.68** (1.12, 2.53)	0.88 (0.52, 1.49)	**1.67** (1.12, 2.50)	0.89 (0.47, 1.71)	1.37 (0.91, 2.04)	1.13 (0.65, 1.96)	1.07 (0.69, 1.67)	**0.52** (0.30, 0.90)	0.53 (0.23, 1.25)	0.73 (0.40, 1.32)	0.59 (0.20, 1.78)
**Race and ethnicity**															
NH African American (Ref)															
NH Asian	1.23 (0.20, 7.60)	0.57 (0.06, 5.58)	2.23 (0.45, 11.09)	<0.001 (<0.001, >999.99)	0.43 (0.07, 2.61)	0.97 (0.08, 11.63)	1.96 (0.40, 9.73)	1.37 (0.11, 16.96)	1.50 (0.31, 7.39)	0.69 (0.06, 7.56)	2.88 (0.56,14.99)	0.47 (0.05, 4.93)	<0.001 (<0.001, >999.99)	1.34 (0.19, 9.55)	<0.001 (<0.001, >999.99)
NH Others	1.90 (0.52, 6.97)	0.68 (0.14, 3.19)	0.53 (0.13, 2.11)	<0.001 (<0.001, >999.99)	1.28 (0.35, 4.71)	<0.001 (<0.001, >999.99)	1.13 (0.31, 4.13)	<0.001 (<0.001, >999.99)	0.56 (0.15, 2.01)	0.91 (0.21, 4.02)	1.95 (0.48, 7.87)	<0.001 (<0.001, >999.99)	<0.001 (<0.001, >999.99)	<0.001 (<0.001, >999.99)	8.85 (0.35, 224.59)
NH White	**2.94** (1.34, 6.41)	1.25 (0.54, 2.91)	0.94 (0.45, 2.00)	1.00 (0.40, 2.47)	1.31 (0.61, 2.83)	2.64 (0.75, 9.32)	1.19 (0.56, 2.51)	1.07 (0.33, 3.52)	0.98 (0.47, 2.05)	0.56 (0.22, 1.39)	1.47 (0.61, 3.56)	0.56 (0.22, 1.43)	1.49 (0.18, 12.43)	1.06 (0.36, 3.10)	0.78 (0.08, 7.42)
Hispanic	**8.03** (2.70,23.8)	**3.76** (1.28,11.09)	2.25 (0.82, 6.17)	2.18 (0.70, 6.82)	1.88 (0.67, 5.26)	**5.69** (1.34, 24.11)	0.55 (0.19, 1.56)	0.14 (0.01, 1.47)	**0.24** (0.08, 0.75)	0.74 (0.23, 2.44)	2.88 (0.96, 8.66)	1.31 (0.41, 4.19)	4.80 (0.48, 48.04)	3.40 (0.97, 11.99)	3.59 (0.33, 39.25)
**Education (highest level completed)**															
Less than high school (Ref)															
Complete HS	0.69 (0.44, 1.10)	0.68 (0.42, 1.09)	0.68 (0.44, 1.07)	0.66 (0.39, 1.11)	**0.62** (0.39, 0.97)	0.61 (0.34, 1.09)	0.73 (0.46, 1.16)	0.92 (0.45, 1.88)	0.85 (0.54, 1.36)	0.85 (0.46, 1.56)	0.65 (0.40, 1.07)	0.80 (0.43, 1.47)	**0.37** (0.15, 0.90)	0.75 (0.39, 1.45)	0.93 (0.29, 2.99)
Some college	0.88 (0.52, 1.49)	0.66 (0.38, 1.16)	**0.52** (0.31, 0.89)	0.81 (0.44, 1.47)	0.78 (0.46, 1.31)	0.95 (0.51, 1.77)	0.71 (0.42, 1.21)	0.95 (0.40, 2.24)	1.07 (0.62, 1.84)	1.34 (0.69, 2.61)	0.70 (0.40, 1.22)	0.54 (0.26, 1.12)	**0.15** (0.04, 0.52)	0.69 (0.33, 1.43)	**0.24** (0.06, 0.92)
College+	**0.23** (0.10, 0.55)	**0.29** (0.11, 0.79)	**0.31** (0.13, 0.74)	0.54 (0.19, 1.58)	0.74 (0.33, 1.68)	0.83 (0.30, 2.28)	0.61 (0.27, 1.39)	0.46 (0.05, 3.95)	0.63 (0.27, 1.49)	0.43 (0.09, 2.10)	**0.34** (0.12, 0.95)	0.70 (0.22, 2.23)	0.20 (0.04, 1.10)	0.46 (0.13, 1.60)	0.24 (0.03, 1.74)
**Occupation status**															
Employed (Ref)															
Unemployed	1.52 (0.82, 2.81)	**1.98** (1.04, 3.76)	1.63 (0.89, 2.98)	1.79 (0.88, 3.66)	1.42 (0.78, 2.60)	0.95 (0.41, 2.20)	0.93 (0.51, 1.71)	1.13 (0.43, 2.93)	0.98 (0.54, 1.78)	**3.03** (1.43, 6.40)	1.72 (0.89, 3.32)	1.16 (0.51, 2.62)	1.01 (0.26, 3.91)	1.69 (0.68, 4.21)	1.91 (0.27, 13.56)
Retired	1.55 (0.86, 2.79)	**2.06** (1.11, 3.83)	**1.96** (1.09, 3.49)	**2.02** (1.01, 4.05)	0.78 (0.42, 1.43)	0.54 (0.21, 1.34)	1.04 (0.58, 1.88)	1.68 (0.67, 4.20)	0.95 (0.53, 1.71)	1.26 (0.52, 3.07)	1.68 (0.86, 3.29)	0.68 (0.27, 1.69)	0.51 (0.11, 2.32)	1.32 (0.50, 3.45)	1.14 (0.11, 11.98)
Full-time homemaker	**1.90** (1.02, 3.53)	1.10 (0.58, 2.11)	1.46 (0.80, 2.67)	1.11 (0.56, 2.23)	1.66 (0.91, 3.03)	1.44 (0.71, 2.94)	1.34 (0.73, 2.45)	0.98 (0.38, 2.58)	**3.02** (1.60, 5.71)	**2.23** (1.07, 4.68)	1.25 (0.65, 2.39)	0.77 (0.32, 1.87)	0.80 (0.24, 2.66)	1.35 (0.57, 3.18)	1.61 (0.38, 6.88)
Student	2.42 (0.49,12.0)	1.34 (0.23, 7.70)	1.46 (0.30, 7.21)	0.61 (0.07, 5.46)	1.40 (0.29, 6.81)	0.52 (0.06, 4.90)	1.92 (0.34, 10.83)	<0.001 (<0.001, >999.99)	1.14 (0.22, 5.81)	2.54 (0.43, 15.10)	1.84 (0.35, 9.65)	3.18 (0.57, 17.64)	**9.33** (1.26, 69.21)	1.11 (0.11, 10.84)	<0.001 (<0.001, >999.99)
**Yearly personal income**	** **	** **	** **	** **	** **	** **	** **	** **	** **	** **	** **	** **	** **	** **	** **
$0–$30K (Ref)															
$31k-$50k	0.66 (0.40,1.09)	1.02 (0.59, 1.78)	0.97 (0.58, 1.61)	1.34 (0.74, 2.43)	1.07 (0.64, 1.77)	1.68 (0.89, 3.17)	1.45 (0.87, 2.42)	0.90 (0.40, 2.04)	0.92 (0.56, 1.53)	1.29 (0.66, 2.52)	1.38 (0.79, 2.41)	0.74 (0.36, 1.52)	0.56 (0.17, 1.83)	0.70 (0.31, 1.57)	**4.49** (1.01, 19.87)
$51k-$100k	1.05 (0.61,1.82)	**1.81** (1.01, 3.23)	1.48 (0.86, 2.55)	1.42 (0.74, 2.70)	1.03 (0.60, 1.79)	1.60 (0.80, 3.21)	1.07 (0.62, 1.85)	1.25 (0.53, 2.98)	1.04 (0.60, 1.82)	1.52 (0.74, 3.15)	1.69 (0.92, 3.09)	0.96 (0.45, 2.07)	1.27 (0.40, 4.00)	1.33 (0.60, 2.93)	**8.92** (1.94, 41.01)
> $100k	0.38 (0.13,1.12)	0.73 (0.21, 2.62)	0.62 (0.21, 1.86)	0.47 (0.10, 2.38)	0.96 (0.36, 2.58)	1.59 (0.47, 5.34)	0.77 (0.29, 2.06)	<0.001 (<0.001, >999.99)	0.52 (0.18, 1.47)	<0.001 (<0.001, >999.99)	1.40 (0.45, 4.30)	0.43 (0.08, 2.25)	0.71 (0.07, 7.02)	1.55 (0.41, 5.88)	<0.001 (<0.001, >999.99)
**Marital status**	** **	** **	** **	** **	** **	** **	** **	** **	** **	** **	** **	** **	** **	** **	** **
Married (Ref)															
Partnered	1.19 (0.60,2.37)	1.35 (0.67, 2.72)	1.36 (0.69, 2.66)	1.03 (0.47, 2.25)	1.13 (0.57, 2.24)	1.53 (0.65, 3.61)	0.96 (0.49, 1.89)	1.42 (0.54, 3.76)	1.33 (0.67, 2.63)	1.08 (0.45, 2.59)	1.29 (0.61, 2.70)	1.45 (0.59, 3.60)	0.71 (0.18, 2.79)	1.07 (0.41, 2.77)	<0.001 (<0.001, >999.99)
Divorced/Separated/Widowed	0.90 (0.55,1.47)	0.90 (0.53, 1.52)	1.19 (0.73, 1.96)	1.09 (0.62, 1.94)	1.20 (0.72, 1.98)	1.78 (0.94, 3.37)	1.09 (0.67, 1.78)	0.99 (0.45, 2.22)	1.32 (0.81, 2.16)	1.37 (0.71, 2.65)	1.47 (0.84, 2.55)	**2.23** (1.12, 4.43)	0.80 (0.28, 2.28)	0.70 (0.31, 1.56)	0.58 (0.11, 3.01)
Single, never married	0.98 (0.56,1.69)	0.85 (0.47, 1.54)	1.22 (0.71, 2.11)	0.65 (0.33, 1.29)	1.05 (0.61, 1.81)	0.93 (0.45, 1.92)	**2.21** (1.27, 3.87)	0.79 (0.33, 1.92)	1.61 (0.93, 2.79)	0.80 (0.38, 1.68)	1.33 (0.74, 2.40)	0.75 (0.34, 1.64)	**0.20** (0.05, 0.82)	0.66 (0.30, 1.48)	0.49 (0.11, 2.15)

Note: Bold numbers represent p<0.05

## Discussion

E-cigarette use is difficult to measure due to the varying habits of users, which often include using the product infrequently, and the many different devices that are used for consumption [23–25]. Our study compared e-cigarette expenditures and 30-day e-cigarette use as measures of the intensity of e-cigarette use. Our findings suggest that expenditures on e-cigarettes may be a useful way of measuring use of the product and that there may be more symptoms associated with e-cigarette use than previously thought. Our results indicate that after controlling for CPD and sociodemographic characteristics, spending on e-cigarettes was positively associated with risks of several disease symptoms. In contrast, there were no statistically significant associations between current e-cigarette use and symptoms. Thus, our findings suggest that spending might be a more useful measure of intensity of e-cigarette use because it captures intensity and frequency of use, as well as multiple types of products used. However, greater expenditures on e-cigarettes could also reflect purchasing more expensive e-liquid or more expensive devices. We were unable to discern this from the data.

Previous studies reported mixed results on the short-term health effects of e-cigarette use among adults. One study collected data on original posts from three online e-cigarette forums and found that 318 negative symptoms were reported by e-cigarette users, including wheezing, shortness of breath, coughing (after using an e-cigarette), sore throat, sensitive teeth, mouth ulcers, headache, fatigue, chest pain, tightening of chest, and cold symptoms [29]. However, another study using an internet survey of e-cigarette and cigarette users found positive health effects, as participants reported improved breathing/respiration, less coughing, fewer sore throats, and reduced bad breath [32]. We did not find a statistically significant association between current e-cigarette use and any of the 15 disease symptoms examined in this study. The possible explanation could be that the binary measure of e-cigarette use is not enough to capture the extent of e-cigarette use while the e-cigarette spending is a continuous variable which can better capture the actual extent of e-cigarette use.

This study has several limitations. First, all data were self-reported and subject to recall bias. Self-report of disease symptoms may produce less accurate prevalence estimates than doctor-diagnosed diseases or biometric data [22]. However, there are several studies which have used self-reported disease symptoms to examine the relationship between e-cigarette use and disease symptoms [16,18]. In addition, self-reported chronic conditions have been shown to have reasonable validity when compared to medical examination-based diagnoses [33,34]. Second, responding affirmatively to the question of current e-cigarette use in the past 30 days might not reflect current e-cigarette use due to recall bias. However, several studies have used past 30-day e-cigarette use to reflect current e-cigarette use [18,26,35]. Third, the survey question did not distinguish daily versus nondaily cigarette smokers, so we were unable to derive CPD for nondaily smokers and treated their CPD as reported. Fourth, we do not know how much of spending on e-cigarettes was for self-use. Fifth, our analysis was based on the first wave of the TABS data, so our estimates do not capture effects over time. Future studies using longitudinal data would allow us to further investigate the causal relationship between spending on e-cigarettes and disease symptoms. Sixth, due to the relatively small sample size, our results may not prove to be robust. Finally, all the people in this study were smokers, so we cannot make any conclusions about the health impacts of sole e-cigarette use alone.

The state of California plans to begin taxing e-cigarettes on an ad valorem basis (i.e. as a percent of value), rather than using an excise tax per pack like the cigarette tax, indicating that they will use expenditures rather than quantity as the basis for the tax [36]. Our findings suggest that spending on e-cigarettes is an appropriate approach for measuring the use of e-cigarettes.

The association of e-cigarette spending and disease symptoms highlights the need to reduce e-cigarette consumption in order to improve health. The 2016 Surgeon General Report has proposed several methods by which e-cigarette use can be reduced, including continuing the regulation of e-cigarettes at the federal level to protect public health, the incorporation of e-cigarettes into smoke-free policies, and the regulation of e-cigarette marketing [33]. Our findings underscore the need for such approaches.

Among adult cigarette smokers who are e-cigarette ever users, those with higher spending on e-cigarettes had greater risks of having chest pain, noticing blood when brushing their teeth, having sores or ulcers in their mouth, and having more than one cold compared to those with lower spending on e-cigarettes. There were no significant associations between 30-day e-cigarette use and disease symptoms. Our findings indicate that e-cigarette expenditure may be a useful proxy for use of e-cigarettes. Finally, the fact that we were able to detect a health effect of e-cigarette use (whether measured by reported use or e-cigarette expenditures) among smokers independent of the effect of the number of cigarettes smoked per day suggests that e-cigarette use adds adverse health effects even among cigarette smokers.

## Supporting information

S1 Data SAS file containing raw data. (SAS7BDAT)
